# ALPK1 phosphorylates myosin IIA modulating TNF-α trafficking in gout flares

**DOI:** 10.1038/srep25740

**Published:** 2016-05-12

**Authors:** Chi-Pin Lee, Shang-Lun Chiang, Albert Min-Shan Ko, Yu-Fan Liu, Che Ma, Chi-Yu Lu, Chung-Ming Huang, Jan-Gowth Chang, Tzer-Min Kuo, Chia-Lin Chen, Eing-Mei Tsai, Ying-Chin Ko

**Affiliations:** 1 Graduate Institute of Medicine, College of Medicine, Kaohsiung Medical University, Kaohsiung, Taiwan; 2 Environment-Omics-Diseases Research Center, China Medical University Hospital, Taichung, Taiwan; 3 Department of Health Risk Management, College of Public Health, China Medical University, Taichung City, Taiwan; 4 Department of Evolutionary Genetics, Max Planck Institute for Evolutionary Anthropology, Leipzig D-04103, Germany; 5 Department of Biomedical Sciences, College of Medicine Sciences and Technology, Chung Shan Medical University, Taichung, Taiwan; 6 Genomics Research Center, Academia Sinica, Taipei, Taiwan; 7 Department of Biochemistry, College of Medicine, Kaohsiung Medical University, Kaohsiung, Taiwan; 8 Graduate Institute of Integrated Medicines, China Medical University, Taichung, Taiwan; 9 Epigenome Research Center, China Medical University Hospital, Taichung, Taiwan; 10 Graduate Institute of Clinical Medical Science, College of Medicine, China Medical University, Taichung, Taiwan

## Abstract

Gout is characterized by the monosodium urate monohydrate (MSU)-induced arthritis. Alpha kinase-1 (ALPK1) has shown to be associated with MSU-induced inflammation and gout. Here, we used bioinformatics, proteomics, cell models, and twenty *in vitro* human assays to clarify some of its role in the inflammatory response to MSU. We found myosin IIA to be a frequent interacting protein partner of ALPK1, binding to its N-terminal and forming a protein complex with calmodulin and F-actin, and that MSU-induced ALPK1 phosphorylated the myosin IIA. A knockdown of endogenous ALPK1 or myosin IIA significantly reduced the MSU-induced secretion of tumour necrosis factor (TNF)-α. Furthermore, all gouty patients expressed higher basal protein levels of ALPK1, myosin IIA, and plasma TNF-α, however those medicated with colchicine has shown reduced myosin IIA and TNF-α but not ALPK1. The findings suggest ALPK1 is a kinase that participates in the regulation of Golgi-derived TNF-α trafficking through myosin IIA phosphorylation in the inflammation of gout. This novel pathway could be blocked at the level of myosin by colchicine in gout treatment.

We previously showed an association for gout in chromosome 4q25 region (OMIM #138900; gout susceptibility 1) using genome-wide familial linkage on ethnic aboriginal Taiwanese known for a high prevalence of tophaceous gout^1^. A region-wide genotyping and population replication attribute the changes to a gene, α-kinase-1 (*ALPK1*) with gout^2^. Although ALPK1 has shown to be expressed alongside proinflammatory cytokines, such as tumour necrosis factor (TNF)-α and interleukin (IL)-1β, via activation of ERK1/2 and p38 in monosodium urate (MSU)-induced human monocytic THP-1 cells^3^, the understanding of biological role of ALPK1 in gouty inflammation remains unclear.

The property of α-kinases includes the phosphorylation of serine and threonine residues on protein substrates containing an α-helix^4^. ALPK1 is known to phosphorylate the tail region of *Dictyostelium discoideum* myosin II^5^ and is implicated in raft-associated sucrase–isomaltase of epithelial cells by phosphorylating the motor protein myosin I that mediates a sorting process via exocytic transport to the apical plasma membrane^6^. ALPK1 is also highly expressed in the thymus and spleen, suggesting a link to the immune system^7^. We hypothesize that ALPK1 participates in the vesicular transport of certain cytokines to the plasma membrane in gout.

In this study, we sought to investigate the ALPK1 protein-binding partners, their interactions, and determine whether cytokine secretion has any mediating effects through phosphorylated myosin in MSU-induced monocytes. We selected IL-1β and TNF-α for detailed study considering that pro-inflammatory cytokines in neutrophil activation of acute gout are IL-1, IL-6, IL-8, TNF-α, macrophage inflammatory protein 1 (MIP-1), monocyte chemoattractant protein-1 (MCP-1)^8^,^9^ and that IL-1β and TNF-α are released by monocytes following an incubation with MSU crystals^10^. To test if ALPK1-associated cytokine response has relevance in gout, we measured its protein level among thirty male individuals: twenty gouty males who had blood drawn during an acute gouty flare in outpatient clinic, and ten healthy males from the same hospital. The two groups were age and gender matched.

## Results

### Prediction of ALPK1 functional domain

**Figure 1:**
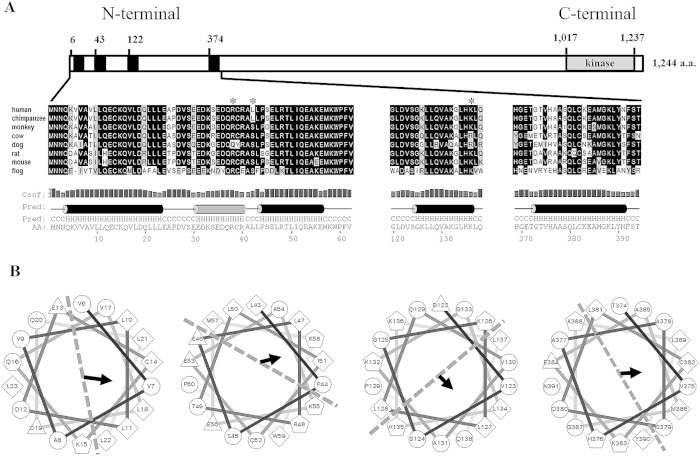
Conserved α-helical structures within ALPK1 N-terminal domain. (**A**) An enlarged view of ALPK1 N-terminal region and cross-species comparison of N-terminal orthologues (asterisks denote highly conserved amino acid residues that have predicted noncovalent properties with myosin IIA); (**B**) N-terminal-conserved α-helical-rich regions of human ALPK1 protein can potentially fold into an amphipathic helical structure, well-suited for vesicular transport and curvature fusion sensors. Here, four putative amphipathic α-helical structures are shown that demonstrated partitions of hydrophobic, nonpolar, negative, and positive residues represented as diamonds, circle, triangles, and pentagons, respectively. The area separated by dotted line illustrates the amphipathic properties and the arrow indicates the hydrophobic moment and direction.

Protein sequence of ALPK1’s N-terminal domain was conserved across several species, such as in human (*Homo* sapiens; NP_001095876), chimpanzee (*Pan troglodytes*; XP_001142586), monkey (*Macaca* mulatta; XP_001093043), cow (*Bos taurus*; XP_0052007724), dog (*Canis* lupus; XP_005639336), rat (Rattus norvegicus; XP_227715), mouse (Mus musculus; NP_082084), and frog (Xenopus tropicalis; NP_001090743) (Fig. 1A). The secondary structure of ALPK1’s N-terminal region was predicted to contain several α-helices, including four putative amphipathic α-helices in the N-terminal region (5–22, 43–60, 88–105, and 122–139 a.a.) of human ALPK1 from helical wheel diagrams (Fig. 1B), and within the C-terminal region, a known α-kinase catalytic domain was present.

### ALPK1 vector construction and protein purification

**Figure 2:**
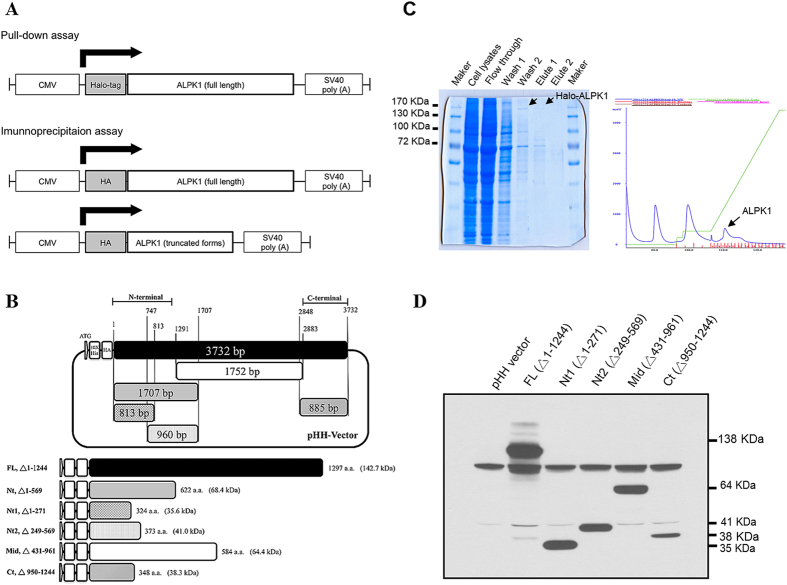
ALPK1 vector construction and confirmation. (**A**) Schema of Halo-ALPK1 full-length and HA-ALPK1 full-length/truncated forms used in pull-down and immunoprecipitation assays. (**B**) Schema of various deletion form constructs (shaded) from a full-length ALPK1 (black), the positions (1–3732 bp) of ALPK1 ORFs are as indicated and residue numbers are base pairs. (**C**) Purification of the Halo-ALPK1 fusion protein using HaloLink resin in mammalian cells, and then pull-down assay using THP-1 cell lysates. Arrows indicate results on the SDS-PAGE gel (left) and wavelength output (right). (**D**) Protein expressions of ALPK1 full-length and deleted form. The HEK293T cells were transfected with full and serial deletion form of the constructed plasmid and confirmed by an anti-HA antibody on Western blot.

In total, one full-length vector with a N-terminal Halo-tag, and six vectors (one full-length and five truncated forms) with a N-terminal HA-tag belonging to ALPK1 were designed and constructed for the pull-down and immunoprecipitation assays (Fig. 2A,B). The recombinant Halo-ALPK1 protein was purified to >90% purity from HEK293F cells, and used in the pull-down assays (Fig. 2C). All the constructs of HA-ALPK1 recombinant proteins were confirmed by Western blotting, and purified for immunoprecipitation assays (Fig. 2D).

### ALPK1 protein binding partners

From the pull-down assay, over 173 ALPK1-binding proteins were identified after five replicates that passed a filter criteria (having ions score >32 indicating identity or extensive homology p < 0.05) and each candidate protein was matched with at least four peptides (detail list in Supplementary Table 1). From the immunoprecipitation assay, 218 cellular proteins interacted with the recombinant ALPK1 produced from one full-length (FL) and five truncated form (Nt, Nt1, Nt2, Mid, Ct) vectors and after three triplicates giving a total of eighteen interactions per candidate protein (Supplementary Table 2). The two assays were conducted to overcome some of limitations during protein–protein interaction, such as a loss of ALPK1-binding proteins during the nickel elution process or a poor affinity of primary antibody for ALPK1. Table 1 shows a summary of the top candidate proteins based on a high frequency of detection in total experiments and sorted according to biological categories. We selected the myosin IIA (having the highest score as well as passing the two assays), and two possible functional associates (F-actin and calmodulin) also appeared in the list that is related to vesicular transport.

### Validation of selected ALPK1 protein binding partners

**Figure 3:**
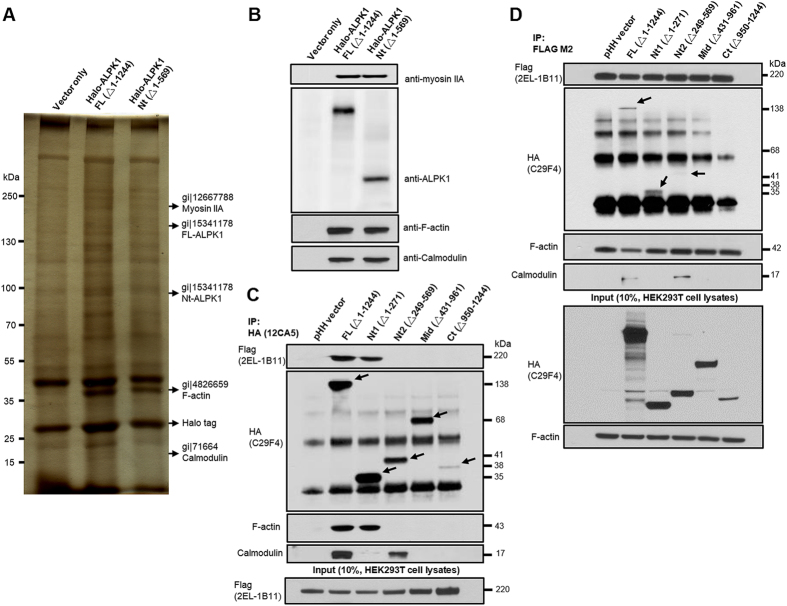
Identification of ALPK1 and three highly scoring protein binding partners: myosin IIA, F-actin, and calmodulin. (**A**) Full-length and N-terminal region of Halo-ALPK1 and halo fusion proteins were overexpressed by transient transfection of HEK293F cells and then purified using HaloLink resin. Arrows indicate protein bands unique to the Halo-ALPK1 lanes [~220, 170, 100, 43, 22 kDa positions were identified as: myosin IIA, full-length (FL)-ALPK1, N-terminal (Nt)-ALPK1, F-actin, calmodulin] by LC/MS/MS protein identification. (**B**) Western blotting using ALPK1, myosin IIA, F-actin, and calmodulin antibodies for the detection of FL-ALPK1, Nt-ALPK1, myosin IIA, F-actin, and calmodulin, and confirmed from the same pull-down assay. (**C**) FL- and Nt1-ALPK1 specifically interacted with both myosin IIA and F-actin. FL- and Nt2-ALPK1 strongly interacted with endogenous calmodulin. Interaction of ALPK1 serial deletion form with myosin IIA, F-actin and calmodulin was confirmed in 293T cells by coimmunoprecipitation with the transfected HA-tagged ALPK1 serial deletion form and Flag-tagged myosin IIA, followed by SDS-PAGE and Western blotting using anti-HA, anti-Flag, anti-F-actin, or anticalmodulin antibodies. (**D**) HEK293T cells were cotransfected with HA-tagged ALPK1 serial deletions form and Flag-myosin IIA. The lysates were subjected to coimmunoprecipitation with an antibody against Flag. Presence of ALPK1, F-actin, and calmodulin in the precipitate was detected using anti-HA, anti-F-actin, and anticalmodulin antibodies.

Transiently transfected populations of HEK293T cells were generated to express recombinant Halo-ALPK1 proteins from the FL- and Nt-constructed vectors for pull-down assays using THP-1 cell lysates. After tandem affinity purification, the colloidal Coomassie staining and SDS-PAGE fractionation were performed. The LC/MS/MS protein identification and Western blotting confirmed the protein bands at 220 kDa (myosin IIA), 43 kDa (F-actin), and 17 kDa (calmodulin) (Fig. 3A,B).

### Identification of myosin IIA binding to ALPK1 N-terminal

Co-immunoprecipitation showed that FL- and Nt1-HA-ALPK1 bound to myosin IIA but not Nt2-, Mid-, or Ct-HA-ALPK1 (Fig. 3C). The FL-, Nt1-, and Nt2-HA-ALPK1 were present in the precipitation of Flag-tagged Myosin IIA in 293T cells, but not mid- or Ct-HA-ALPK1 (Fig. 3D). The inconsistency between both precipitation assays was the Nt2-HA-ALPK1 truncated form. It indicated to us that towards the Nt1 region of ALPK1 contained a binding site for myosin IIA. Similarly, the FL- and Nt2-HA-ALPK1 strongly interacted with endogenous calmodulin while Nt1-HA-ALPK1 with calmodulin was weak (Fig. 3C,D). The immunoprecipitation Flag-tagged myosin IIA detected F-actin, and F-actin interacted with FL- and Nt1-HA-ALPK1. The results indicated that the binding complexes of myosin IIA, F-actin, and calmodulin were most likely to locate in the N-terminal of ALPK1.

### MSU-induced phosphorylation of myosin IIA is ALPK1 dependent

**Figure 4:**
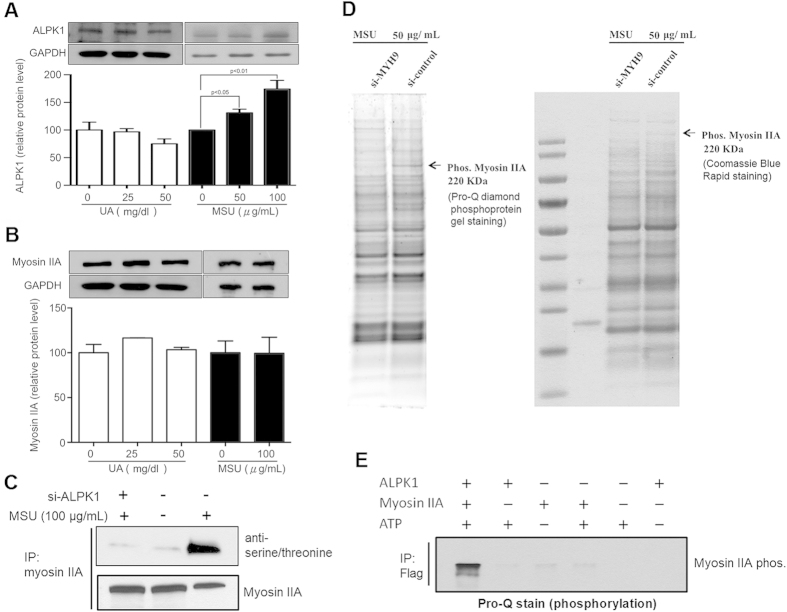
MSU-induced phosphorylation of myosin IIA is ALPK1 dependent. (**A**) After 16-h treatments with uric acid (0, 25, and 50 mg/dl) or MSU crystals (0, 50, and 100 μg/mL), uric acid did not increase the protein expression of ALPK1, however MSU did (p < 0.05). (**B**) Uric acid and MSU did not affect myosin IIA expression. (**C**) Both MSU treatment and ALPK1 increased myosin IIA phosphorylation (lane 3) in THP-1 cells using antiphospho-serine/threonine antibody. (**D**) Phosphorylated myosin IIA was confirmed at the 220-kDa position, from comparing the depletion and no depletion of myosin IIA by MYH9 siRNA, on the Pro-Q staining SDS-PAGE gel (left) and protein-specific Coomassie Blue staining gel (right). A control without MSU stimulus is shown in Fig. S3. (**E**) Without MSU stimulus, the phosphorylation of myosin IIA is shown to require ALPK1 and ATP (lane 1) on the Pro-Q staining assay. The weak myosin IIA bands for example in second lane that has no myosin were trace myosin IIA bound to ALPK1 FL and Nt1, refer to Fig. 3C, from purification that was co-eluted.

Figure 4A shows the THP-1 cells treated with uric acid in a soluble form did not significantly influence ALPK1 expression. However, after a 16-h treatment of MSU crystals (0, 50, 100 μg/mL) in THP-1 cells, ALPK1 was significantly induced compared to untreated cells (p < 0.05). No effect was observed for the expression of endogenous myosin IIA after stimulation with uric acid (50 mg/dl) or MSU crystals (100 μg/mL; Fig. 4B). Figure 4C shows that using antiphospho serine/threonine antibody, ALPK1 had increased the phosphorylation of myosin IIA in MSU-treated THP-1 cells. Figure 4D shows that a phosphorylated myosin IIA (220 kDa) could not be detected on the Pro-Q stained SDS-PAGE gel and protein-specific Coomassie Blue stained gel when the myosin IIA was knocked down by *MYH9* siRNA in the 16-h MSU treated THP-1 cells (Fig. 4D). To see if ALPK1 phosphorylated myosin IIA in HEK293 cells without MSU, an *in vitro* kinase assay was prepared. The phosphorylation of myosin IIA was strongly seen after incubation with both ALPK1 and ATP on the Pro-Q staining assay (Fig. 4E). A weak myosin IIA band in the second lane that does contain myosin IIA was an artefact of purification, as some ALPK1 recombinant protein from HEK 293F cells by Halo-tag system that ALPK1 remained bound to endogenous myosin IIA were co-eluted (Fig. 4E).

### ALPK1 and myosin IIA is related to secretion of MSU-induced cytokines

**Figure 5:**
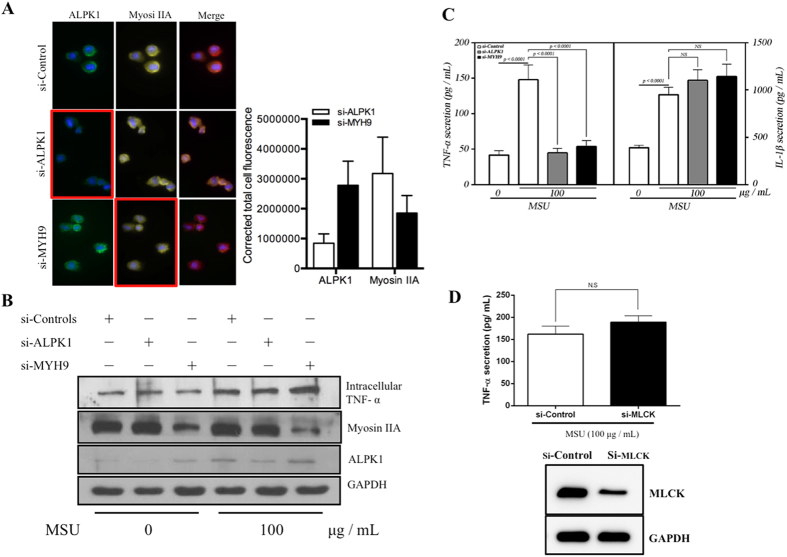
TNF-α secretion is relatively controlled by ALPK1 and myosin IIA in MSU-induced monocytes. (**A**) Protein knockdown of si-ALPK1 and si-MYH9 by immunofluorescence (left) and quantification of intensity of colour (right) showed they were effective but not complete knockdown. (**B**) MSU treatment (compared to none) has generally increased intracellular TNF-α levels, including when ALPK1 was knockdown, which could be explained by the limitation in knockdown efficiency (see Fig. 5 **A**) or other protein kinases that phosphorylated myosin IIA were not eliminated. The most intracellular TNF-α increase was seen with myosin IIA knockdown. (**C**) MSU-induced TNF-α secretion was reduced with the knockdown of the endogenous ALPK1 or myosin IIA, however no effect was observed for IL-1β secretion. (**D**) TNF-α secretion was unaffected by the depletion of another kinase that may phosphorylate myosin IIA, myosin light-chain kinase (MLCK), using siRNA for 24 h prior treatment with MSU. Data are representative of results performed in triplicates. *p < 0.0001.

Immuno-cytofluorescence microscopy showed siRNAs of *ALPK1* and *MYH9* have led to a depletion of endogenous ALPK1 and myosin IIA in THP-1 cells (Fig. 5A). Intracellular TNF-α has increased after MSU treatment (100 μg/mL) and most effects came from the knockdown of myosin IIA (Fig. 5B). Extracellular TNF-α secretion was significantly reduced by the knockdown assays of endogenous ALPK1 and myosin IIA but not IL-1β (Fig. 5C) thus IL-1β was not followed through into the human assays of gout patients. We compared ALPK1 to myosin light-chain kinase (MLCK), a known serine/threonine-specific protein kinase that phosphorylates the regulatory light chain of myosin II, however we found no change to the TNF-α secretion after MLCK knockdown in THP-1 cells for 24-h prior to MSU treatment (100 μg/mL; Fig. 5D).

### Higher levels of ALPK1, myosin IIA, and plasma TNF-α in gouty patients

**Figure 6:**
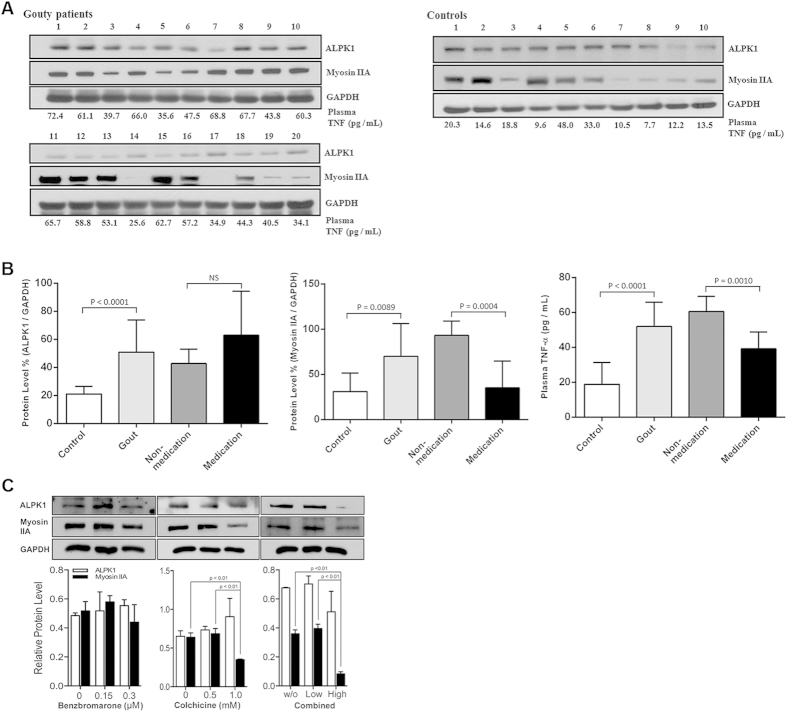
Gouty patients express higher protein levels of ALPK1, myosin, and plasma TNF-α. (**A**) *In vitro* human assays of freshly isolated monocytes from twenty gout patients and ten healthy controls were analysed for the levels of ALPK1, myosin IIA, and plasma TNF-α. Eight gout patients (no. 3, 5 12, 14, 17, 18, 19, 20; details refer to in Supplementary Table 3) who were medicated with a combined benzbromarone and colchicine showed lower plasma TNF-α than the remaining twelve gout patients who were nonmdicated. (**B**) Gout patients were pooled to show they expressed more ALPK1, however no difference was observed between medicated and nonmedicated groups (left), gout patients expressed more myosin IIA, which was reduced in the medicated group (central), gout patients have higher levels of plasma TNF-α (right), especially in the nonmedicated (60.5 ± 8.7 pg/mL) than medicated patients (39.2 ± 9.7 pg/mL) or healthy controls (18.8 ± 12.6 pg/mL). (**C**) *In vitro* effect of medication (benzbromarone, colchicine, or combined of two medications) showed a high dose colchicine (1 mM) had reduced myosin IIA. There was no effect on ALPK1 expression. The THP-1 cells had been incubated with 100 nM PMA for 3 h and then treated with 100 μg/ml MSU for 16 h.

Figure 6A shows twenty gout patients were further categorized into 8 medicated (treated with benzbromarone and colchicine) intercurrent gout patients, and 12 nonmedicated gout patients (flares cases). The endogenous ALPK1 and myosin IIA levels were quantified by GAPDH from purified monocytes using an immunoblotting assay, and plasma TNF-α determined by ELISA. We found endogenous ALPK1, myosin IIA, and plasma TNF-α were higher among gout patients than healthy controls (p < 0.01; Fig. 6B). No significant difference was detected for ALPK1 between the medicated and nonmedicated groups, however myosin IIA (p = 0.0004) and plasma TNF-α levels (p = 0.001) were reduced in response to medication (Fig. 6B).

### Colchicine downregulates myosin IIA in THP-1 cells

Figure 6C shows a high dose colchicine (1 mM) reduced the protein level of myosin IIA in MSU-stimulated THP-1 cells (adherent macrophages), but there was no noticeable effect on ALPK1. Treatment with benzbromarone alone did not affect either myosin IIA or ALPK1. However, a combined treatment of these two medications in MSU-stimulated THP-1 cells resulted in a highly effective reduction (75%) of myosin IIA protein level.

## Discussion

**Figure 7:**
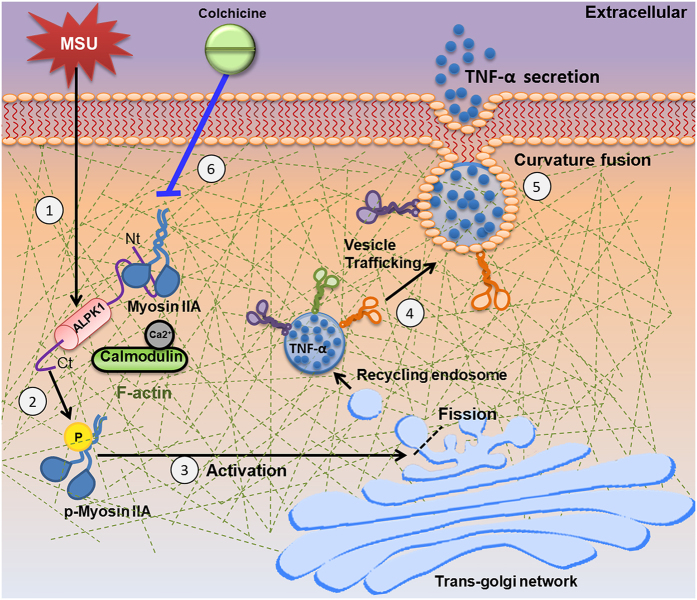
Possible role of ALPK1 in gouty inflammation. (1) MSU crystals stimulate *ALPK1* gene overexpression in the monocyte. (2) One of α-kinase’s functions is bind with myosin IIA via N-terminal (Nt) while C-terminal (Ct) phosphorylates the motor protein in presence of ATP. (3) Activated myosin IIA at the Golgi membrane. (4) Transport of Golgi-derived TNF-α vesicles. (5) Transport towards plasma membrane and contribute to secretion of TNF-α. (6) ALPK1 is unaffected by colchicine, so MSU persists in stimulating *ALPK1* expression. However, colchicine (blue line) disrupts microtubule formation, which myosin motor proteins act upon, thus less myosin is recruited, resulting in less vesicular delivery of TNF-α. An aberrant ALPK1 or knockdown may decrease phosphorylation of myosin IIA and lower TNF-α secretion.

We found ALPK1 phosphorylation of non-muscle myosin IIA was relatively specific for the MSU-induced secretion of TNF-α. The role of ALPK1 in gouty inflammation could be one of many cellular responses to MSU (Fig. 7). Briefly, we purified ALPK1 and demonstrated that myosin IIA was a frequent protein-binding partner, and that MSU-induced ALPK1 phosphorylated myosin IIA, which were abrogated with ALPK1 knockdown by siRNA. We inferred that ALPK1 and myosin IIA might be specific for Golgi-derived transport of TNF-α, because the knockdown of ALPK1 and myosin IIA affected the secretion of TNF-α. The secretion of IL-1β was unaffected, which fitted with the notion that TNF-α and IL-1β are dependent and independent of Golgi-trafficking, respectively^11^. A limitation is that we have not excluded all the protein kinases that could phosphorylate myosin IIA, however the knockdown of another kinase, MLCK, did not appear to affect MSU-induced secretion of TNF-α. Lastly, *in vitro* human assay showed that colchicine inhibited myosin IIA but not ALPK1, indicating that some of MSU-induced TNF-α pathway could be blocked.

ALPK1 is a member of α-kinase family sharing an evolutionary relationship to conventional eukaryotic serine/threonine/tyrosine protein kinase superfamily^12^. While predicting the ALPK1 structure, we observed that the N-terminal region had several conserved secondary α-helix structures shown on helical wheel diagrams with amphipathic properties (hydrophobic and hydrophilic structure distributions), which described a functional potential of the N-terminal helical conformation for curvature membrane sensing and vesicular trafficking. We proceeded to construct the ALPK1 full-length and truncated vectors and then used pull-down and immunoprecipitation to identify myosin IIA as one of the significant proteins binding to ALPK1. The pull-down assay also showed an interaction with calmodulin and F-actin. The direct and indirect bindings in co-immunoprecipitation assays have characterized the protein complexes with ALPK1 to be myosin IIA, calmodulin, and F-actin. The complex assembly primarily occurred at the N-terminal region of ALPK1 (residue 1–569 a.a). The myosin IIA was bound to ALPK1, especially at the Nt1 segment (1–271 a.a.), which matched with a higher density of α-helices predicted binding sites towards the N-terminal end (Fig. 1A). Calmodulin was bound to the Nt2 segment (249–569 a.a.). A study has indicated that Ca^2+^ and calmodulin reduced the myosin IA phosphorylation by calmodulin inhibition^6^. It is likely that Ca^2+^/calmodulin is necessary for ALPK1 to catalyse the phosphorylation of myosin IIA. Peripheral bundles of myosin IIA and F-actin were found with the co-precipitated Flag-myosin IIA confirming that myosin II-mediated contraction of actin filament assembly occurs via its crosslinking properties^13^,^14^,^15^.

The role of myosin II, as a vesicle transport through the actin cytoskeleton, includes the fission of transport carriers from the transGolgi network (TGN) membrane^16^,^17^,^18^, the maintenance of an open fusion pore at plasma membrane^19^,^20^,^21^, and the formation of recycling endosomes through MLCK phosphorylation^22^. Studies indicate activated myosin II can initiate a binding to F-actin^23^,^24^ via the phosphorylation of regulatory light chain subunits by calcium-dependent MLCK^25^. Myosin IIA has also been studied in a variety of immune responses^26^, for example, it is required in a critical step between natural killer immunological synapse formation and granule exocytosis^14^. Although our results implicated myosin IIA in MSU-induced TNF-α secretion, it should be mentioned that myosin IIA is among the multiple of GTPases and other myosins that are implicated in the TGN-to-plasma membrane transport, such as Rab protein 6, 8, and 11a^11^,^17^, and myosins Ic, Ie, II, V, and VI^20^,^27^,^28^.

Despite the flag-myosin IIA was not observed with the ALPK1 C-terminal on Western blot, it does not exclude alpha kinase from being involved with the phosphorylation of myosin IIA. The ALPK1 C-terminal is a kinase domain is very similar to that of TRPM7^29^,^30^, and the α-kinase domain of TRPM7 plays a critical role in myosin II, including regulation of the filament assembly^31^ and stability^32^. Structurally, the kinase domain may act as a dynamic molecular switch, transiently phosphorylating the substrate and then leaving the donor. One switch is the activation segment typically ordered by a dynamic phosphorylation event, as we have demonstrated (Figs 4C,D and 5D), further requiring ATP-activation (Fig. 4E), and the other is a helical subdomain that provides docking sites for protein/peptide substrates^33^.

Lastly the *in vitro* human assay, all gouty patients expressed higher basal protein levels of ALPK1, myosin IIA, and plasma TNF-α than healthy controls after adjusting for age. TNF-α is higher in patients with gout^34^,^35^,^36^ and TNF-α blocking with etanercept (anti-TNF-α) has been developed for gout management^37^. We examined two medications commonly prescribed for gout, benzbromarone that suppress uric acid reabsorption at renal tubules and colchicine that disrupts microtubule assembly^38^, to see if they affected ALPK1, myosin IIA, and TNF-α levels. The myosin IIA and TNF-α were reduced in human assays, and further *in vitro* THP-cells confirmed high dose 1 mM colchicine inhibited myosin IIA, which may be a mechanism contributing to its toxicity in a lethal overdose^39^. Possibly, the colchicine treatment inhibited the apical movements of myosin II through inhibition of the microtubule depolymerisation^40^. The microtubule disruption led to less recruitment of myosin II and TNF-α able to be secreted.

In conclusion, we integrated bioinformatics and proteomic approaches to construct and purify the ALPK1 vectors, which interacted with myosin IIA, F-actin, calmodulin. A MSU-dependent ALPK1 phosphorylation of myosin motor protein could enable the vesicle trafficking of TNF-α. Conceivably, an aberrant ALPK1 might delay normal cytokine response and contribute to chronic gout and tophi, as these were observations in the original affected population^1^. The results from *in vitro* monocytes and human assays corroborate and allow for some new understanding in the pathogenesis of gout.

## Materials and Methods

### Bioinformatics analysis and vector construction

The conserved ALPK1 secondary structure and possible functional domain were predicted by the PSIPRED Protein Sequence Analysis Workbench (v3.3, http://bioinf.cs.ucl.ac.uk/psipred/)^41^, helical wheel diagram analysis^42^ and predicting binding site of proteins^43^. The helical wheel plot was generated using the Perl-based application at http://rzlab.ucr.edu/scripts/wheel/wheel.cgi. The C-terminal region and several amphipathic helices in the N-terminal region of ALPK1, a full length of 1244 amino acids (a.a.) and an N-terminal fragment of 569 a.a. of the human *ALPK1* cDNA sequence containing an N-terminal Halo-tag were constructed into the pFN21k vector (Promega, Madison, WI). Based on predicted regions of interest, we constructed five vectors expressing the ALPK1 protein with an N-terminal 10X His-tag and HA-tag, including full-length (FL, 1–1244 a.a.) and truncated forms of N-terminal (Nt, Nt1, and Nt2; 1–569, 1–271, and 249–569 a.a., respectively), middle (mid; 432–961 a.a.), and C-terminal regions (Ct; 950–1244 a.a.). The full length of the MYH9 (myosin IIA) cDNA sequence (1–1960 a.a.) containing an N-terminal 3X FLAG-tag and 6X His-tag was also constructed (pFH-G2-MYH9). The cDNA sequence of all constructs was verified by Sanger DNA sequencing.

### Cell culture and transfection

Suspension-adapted FreeStyle 293F cells (Invitrogen, Carlsbad, CA) were routinely cultured in GIBCO FreeStyle 293F expression medium in disposable Erlenmeyer tissue culture fl293F with vented caps (Corning Inc., Corning, NY) at 120 rpm on an orbital shaker platform (MIR-S100C shakers with universal platforms, SNAYO Electric). These cells were kept in a cell culture incubator (37 °C, 8% CO_2_). In transient transfections, 50 μg of vector DNA diluted in OptiPro SFM (Gibco) was gently mixed with 40 μL of FreeStyle MAX Reagent (Invitrogen) diluted in OptiPro SFM and then incubated for 10 min at room temperature. The mixture was added dropwise to 40 mL of 293F cells in a flask, and the transfected cells were allowed to grow in suspension. After 48 h, cells were separated from the Halo-ALPK1 293F-containing supernatant by centrifugation and stored at −80 °C until use. HEK293T cells were maintained in α-MEM (Gibco) with 10% FBS (Gibco) and 1% penicillin–streptomycin (Gibco). HEK293T cells were transiently cotransfected with plasmids coding for FLAG-tagged myosin IIA and HA-tagged full-length ALPK1 or serial deletion forms and the corresponding control plasmids in six-well plates. Briefly, 4 μg of vector DNA and 8 μL of Lipofectamine 2000 (Invitrogen) were used for transient transfection. The transfected HEK293T cells were cultured under standard conditions (5% CO_2_ and 37 °C) for 24 h prior to harvest.

Human monocytic THP-1 cells were obtained from ATCC, and maintained at 2 × 10^5^ cells/ml in RPMI 1640 medium supplemented with 10% FCS and 2 mmol/L L-glutamine. THP-1 cells were induced to differentiate into adherent macrophage-like cells by treatment with 100 nm phorbol 12-myristate 13-acetate (PMA) (Sigma-Aldrich, St. Louis, MO, USA) for 3 hours. In different sets of experiments, the culture medium was replaced with medium lacked serum (RPMI with 1% fetal bovine serum) for 15 min prior to MSU treatments (0, 50, and 100 μg/mL) or uric acid treatments (0, 25, and 50 mg/dL) for 24 h^44^, respectively. Experiments investigating the effects of pharmacological inhibitors in MSU-treated THP-1 cells for 16 hour, followed by 6 hours treatment with, without or both benzbromarone (0, 0.15, and 0.3 μM)^45^ and colchicine (0, 0.5, and 1 mM)^46^.

### Preparation of MSU crystals

MSU was prepared as previously described^3^. The culture medium was replaced with serum-free medium for 15 min prior to MSU treatments (0, 50, and 100 μg/mL; Supplementary Figure 1).

### Halo-tagged proteins and pull-down assays

Halo-ALPK1 full-length (FL) and N-terminal (Nt) recombinant proteins were expressed in FreeStyle 293F cells. Cells were lysed using a 27-gauge needle in cell lysis buffer [50-mM HEPES (pH 7.5), 150-mM NaCl, 0.5-mM EDTA, 0.1% Na deoxycholate, and a Roche complete protease inhibitor cocktail]. The cell lysate was centrifuged to remove debris, and the supernatant was coupled to the HaloLink resin (Promega) by incubation at 4 °C for 6 h. The HaloLink resin was added at a ratio of 1 mL of resin per 5 mL of cleared cell lysate. The resin was washed extensively with Halo-tag protein purification buffer [50-mM HEPES (pH 7.5) supplemented with 1-mM DTT, 320-mM sucrose, 0.005% IGEPAL^®^ CA-630 (Sigma)]. To check coupling efficiency, an aliquot of the resin was resuspended in Halo-tag protein purification buffer containing 0.1 μg/μL of tobacco ETCH virus (TEV) protease to release covalently bound ALPK1 FL or Nt bait protein and was visualized by SDS-PAGE following Coomassie Blue staining.

Pull-down assay was performed according to the modified protocol of the Halo-Tag Mammalian Pull-down system (Promega). Recombinant fusion proteins of ALPK1 FL and Nt were expressed and purified from 293F using the HaloTag Protein Purification System. Briefly, 300 μg of THP-1 protein lysate was incubated with Halo-tagged fusion proteins overnight. Samples were then incubated with HaloLink resin at 4 °C for 4 h before washing with lysis buffer supplemented with 0.01% tween-20 and elution by TEV protease cleavage. The protein profiling of samples from the empty vector and ALPK1 FL and Nt were loaded on 7.5% SDS-PAGE gels with equal amounts and observed by silver staining (Invitrogen) or Western blotting. The pull-down was conducted in parallel with control beads. UPLC-MS/MS was used to further identify eluted proteins. Protein–protein interaction screening was accomplished in five independent experiments.

### In-solution digestion and protein identification

A total of 50 μL of protein solution was thoroughly mixed with 500 μL of acetone following centrifugation at 13,000 rpm for 10 min. The protein residues were evaporated to dryness after discarding the supernatant. Protein residues were redissolved with 25 mM of ammonium bicarbonate aqueous solution and digested with sequence-grade trypsin (Promega) at 37 °C for 16 h. Subsequently, 2 μL of tryptic peptide solution was injected into the nanoACQUITY UPLC system (Waters, Milford, MA, USA) containing a desalting column (Symmetry C18, 5 μm, 180 μm × 20 mm) and an analytical column (BEH C18, 1.7 μm, 75 μm × 100 mm) and was detected by an LTQ Orbitrap Discovery Hybrid fourier transform mass spectrometer (Thermo Fisher Scientific Inc., Bremen, Germany) at a resolution of 30000 coupled with a nanospray source in a positive ion mode. Individual raw data were processed using Mascot Distiller software (Version 2.2, Matrix Science Inc., Boston, MA) and uploaded to the in-house Mascot server for protein identification.

### Co-immunoprecipitation and Western blotting

For immunoprecipitation, 30 μg of total protein from transfected HEK 293 cells was incubated with HA (12CA5, Roche) or Flag (M2, Sigma) antibodies in 50 μL of IP buffer at 4 °C for 4 h. Protein G Mag sepharose (Dynabeads, Invitrogen) were added to the mixture and incubated overnight. The complex was placed on a magnet and washed three times. Proteins eluted from the sepharose beads were subjected to SDS-PAGE and immunoblotting using HA (C29F4, Cell Signaling) or Flag (2EL-1B11, Merck Millipore) antibodies. For sequential reprobing of the same blots, the membranes were stripped and hybridized with another primary antibody. Blots were developed using an enhanced chemiluminescence detection kit (Amersham) and protein intensities were quantified using Image J software (version 1.48, http://imagej.nih.gov/ij/).

### RNA interference

Negative control, ALPK1 (s37074), and MYH9 (s224) Stealth® siRNAs duplex were purchased from Invitrogen. SiRNAs were transiently transfected into THP-1 cell lines using Lipofectamine® RNAiMAX Transfection Reagent (Invitrogen) according to the manufacturer’s instructions.

### Kinase assay

The vector with FLAG-tagged myosin IIA was transfected into HEK293T cells for 24 h, and then the recombinant protein FLAG-tagged myosin IIA was captured and purified from total protein lysate by immunoprecipitation. Samples were resuspended in 200 μL of phosphatase buffer containing two units of phosphatase (Roche) and incubated at 37 °C for 1 h. The phosphatase was then inactivated at 75 °C for 5 min. The immunocomplex was incubated at 30 °C for 30 min in 20 μL of kinase assay buffer containing 1-mM ATP and 1 μg of purified recombinant ALPK1. Separation of phosphorylated and nonphosphorylated myosin IIA was performed by NuPAGE Bis-Tris gradient gel (Invitrogen) following Coomassie Blue Rapid staining (Bioman) or Pro-Q diamond phosphoprotein gel staining (Invitrogen) according to the manufacturer’s instructions. The phosphoprotein staining gel was imaged on a typhoon 9400^TM^ (GE Healthcare, USA) using an excitation wavelength of 532 nm and LPG filter. Myosin IIA was co-precipitated with the cell lysate of HEK293T and dephosphorylated by phosphatase. The proteins that were bound to the antibody were then incubated with or without purified recombinant ALPK1, substrates (myosin IIA) and ATP.

To perform the *in vivo* kinase assay, either myosin IIA siRNA or negative control siRNA was transfected into THP-1 cells. After transfection, the cells were treated with MSU at 37 °C for 16 h in the phosphate-free media containing 10% of dialysed FBS. The samples were precipitated using the methanol–chloroform–water method. The precipitated samples were dissolved in 50 μL of lysis buffer [8-M Urea, 30-mM Tris-HCl, 4% CHAPS (pH 8.5)]^47^. The supernatants were separated on 8% SDS-PAGE gel following Coomassie brilliant blue staining and Pro-Q Diamond phosphoprotein gel staining. In addition, transient transfection was performed using siALPK1 and then treated with MSU at 37 °C for 16 h. The immune complex was separated using Western blotting, and phosphorylation was determined using an antiserine/threonine antibody. A parallel set of myosin IIA was assessed by Western blotting to confirm and compare the level of myosin IIA in a similar amount of total proteins.

### siRNA validation efficiency using immunofluorescence

THP-1 cells were cotransfected with si-ALPK1 and/or si-MYH9. The harvested cells were rinsed with 1 × PBS twice and fixed with 4% paraformaldehyde for 5 min. The fixed cells were spread on coverslips following air-drying for immunofluorescence analysis. Slides were permeabilized with cold methanol at −20 °C for 20 min and then blocked with 10% goat serum containing 0.05% of Triton X-100 at 25 °C for 1 h. Subsequently, the slides were hybridized with anti-ALPK1 and myosin IIA antibodies (1:100) overnight. After rinsing with PBS, slides were incubated with fluorescein-conjugated secondary antibody (1:1000, Invitrogen). The coverslips were mounted with ProLong® Gold antifade reagent containing DAPI and visualized under an automated upright microscope system (Leica DM4000 B, Leica Microsystems). The method for measuring cell fluorescence using ImageJ is following with previous study^48^.

### *In vitro* protein assay of human samples

We recruited 20 male gout patients diagnosed by clinical rheumatologists at the Department of Immunology and Rheumatology, China Medical University Hospital, according to the criteria of the American College of Rheumatology^49^. From the same hospital, 10 male healthy controls, without diagnosis of gout or taking any treatment with hypouricemic agents, were recruited from the Health Examination Center. Written informed consent was obtained from all participants. The Institutional Review Board of China Medical University Hospital (CMUH102-REC1-005) has approved this study. The clinical characteristics are shown in Supplementary Table 3. The gout patients were assigned into medicated and nonmedicated groups. The medicated group (six chronic patients and two acute patients) were administered benzbromarone and colchicine before blood drawn. The nonmedicated patients (eight acute patients and four chronic patients) were not medicated before blood drawn. The age of mean and standard deviation of the two groups were 50.2 ± 16.6 and 53.9 ± 13.0 years old, and not statistically different (t-test p-value = 0.54). The peripheral blood monocytes were isolated from heparinized blood using Ficoll-Hypaque PLUS solution (Amersham Biosciences) and purified using a Dynabeads® CD14 system (Life Technologies, Carlsbad, CA; Supplementary Figure 2). Plasma TNF-α of all volunteers was measured using Human TNF-α ELISA kit (R&D, Plymouth Meeting, PA) according to the manufacturer’s instructions.

### Statistical analysis

Results are expressed as mean ± SD. The statistical analysis between the two groups was assessed using the Mann–Whitney *U* test. A p-value <0.05 was considered statistically significant. General linear model (GLM) was used to compare the ALPK1 and myosin IIA expression levels with medication status and adjusted for age.

## Additional Information

**How to cite this article**: Lee, C.-P. *et al.* ALPK1 phosphorylates myosin IIA modulating TNF-α trafficking in gout flares. *Sci. Rep.* **6**, 25740; doi: 10.1038/srep25740 (2016).

## Supplementary Material

Supplementary Information

## Figures and Tables

**Table 1:** Top scoring ALPK1 protein binding partners from pull-down and immunoprecipitation assays.

Biological function	ID	Protein name	Pull-down		Immunoprecipitation†						
			Passed (%)	Score*	FL	Nt	Nt1	Nt2	Mid	Ct	Passed (%)
**Motor protein**	gi|12667788	Myosin-9	5/5 (100%)	2067	3	3	3	2	0	2	13/18 (72%)
	gi|1841430	Heavy neurofilament subunit	5/5 (100%)	1925	2	1	2	0	2	3	10/18 (56%)
	gi|5453740	Myosin regulatory light chain 12A	4/5 (80%)	175	2	0	1	1	1	1	6/18 (33%)
	gi|190281	Protein phosphatase I alpha subunit partial	5/5 (100%)	814	1	0	0	0	0	1	2/18 (11%)
**Trafficking**	gi|4826659	F-actin-capping protein subunit beta isoform 1	5/5 (100%)	1388	3	3	1	0	0	0	7/18 (39%)
	gi|4502249	Arf-GAP with SH3 domain, ANK repeat and PH domain-containing protein 2 isoform a	5/5 (100%)	1881	2	2	1	1	0	1	7/18 (39%)
	gi|71664	Calmodulin	5/5 (100%)	1622	2	2	2	0	0	0	6/18 (33%)
	gi|4520225	Rho kinase	5/5 (100%)	1456	0	1	1	0	0	0	2/18 (11%)
**Cytoskeleton**	gi|134133226	POTE ankyrin domain family member E	5/5 (100%)	1070	1	1	2	0	1	1	6/18 (33%)
	gi|6759917	A-kinase anchoring protein 18 gamma	5/5 (100%)	1508	1	0	0	0	1	1	3/18 (17%)
	gi|188586	Myosin light chain 2	4/5 (80%)	580	0	0	1	1	1	0	3/18 (17%)
**Golgi-resident**	gi|3641621	gp180-carboxypeptidase D-like enzyme	5/5 (100%)	1937	3	2	2	1	2	2	12/18 (67%)
**Vesicular**	gi|7656922	Charged multivesicular body protein 2a	5/5 (100%)	1705	2	0	1	1	1	1	6/18 (33%)

^*^LC/MS/MS mean identification score.

^†^Abbreviation of ALPK1 full-length (FL) and truncated forms, see text; for extended list, see Supplemental Table 1 and 2.
