# Corrigendum: ALPK1 phosphorylates myosin IIA modulating TNF-α trafficking in gout flares

**DOI:** 10.1038/srep27323

**Published:** 2016-06-10

**Authors:** Chi-Pin Lee, Shang-Lun Chiang, Albert Min-Shan Ko, Yu-Fan Liu, Che Ma, Chi-Yu Lu, Chung-Ming Huang, Jan-Gowth Chang, Tzer-Min Kuo, Chia-Lin Chen, Eing-Mei Tsai, Ying-Chin Ko

Scientific Reports 6: Article number: 2574010.1038/srep25740; published online: 05122016; updated: 06102016.

In this Article, Chia-Lin Chen is incorrectly affiliated with ‘Department of Biomedical Sciences, College of Medicine Sciences and Technology, Chung Shan Medical University, Taichung, Taiwan’. The correct affiliation is listed below:

Genomics Research Center, Academia Sinica, Taipei, Taiwan.

