# Supplementary material for: ALPK1 phosphorylates myosin IIA modulating TNF-α trafficking in gout flares
Source: Sci Rep. 2016 May 12;6:25740. doi: 10.1038/srep25740 (PMC4864424; doi:10.1038/srep25740)
Supplement: Supplementary Information [file srep25740-s1.pdf]

ALPK1 phosphorylates myosin IIA modulating TNF-α trafficking in gout flares

Chi-Pin Lee[1], Shang-Lun Chiang[2, 3], Albert Min-Shan Ko[4], Yu-Fan Liu[5], Che Ma[6], Chi-Yu Lu[7], Chung-Ming Huang[8], Jan-Gowth Chang[9], Tzer-Min Kuo[2], Chia-Lin Chen [5], Eing-Mei Tsai[1], Ying-Chin Ko[2, 10]*

[1]Graduate Institute of Medicine, College of Medicine, Kaohsiung Medical University, Kaohsiung, Taiwan

[2]Environment-Omics-Diseases Research Center, China Medical University Hospital, Taichung, Taiwan

[3]Department of Health Risk Management, College of Public Health, China Medical University

[4]Department of Evolutionary Genetics, Max Planck Institute for Evolutionary Anthropology, Leipzig D-04103, Germany

[5]Department of Biomedical Sciences, College of Medicine Sciences and Technology, Chung Shan Medical University, Taichung, Taiwan

[6]Genomics Research Center, Academia Sinica, Taipei, Taiwan

[7]Department of Biochemistry, College of Medicine, Kaohsiung Medical University, Kaohsiung, Taiwan

[8] Graduate Institute of Integrated Medicines, China Medical University, Taichung, Taiwan

[9] Epigenome Research Center, China Medical University Hospital, Taichung, Taiwan.

[10]Graduate Institute of Clinical Medical Science, College of Medicine, China Medical University, Taichung, Taiwan

*Correspondence: Ying-Chin Ko, E-mail: ycko0406@gmail.com and ycko@mail.cmu.edu.tw; TEL 886-4-2205-2121 ext.7621.
China Medical University, 2 Yude Road, Taichung, 40447, Taiwan (ROC)

Supplementary Table1

ID	Gene name	Passed*	Score
gi|12667788	myosin-9 [Homo sapiens]	5/5	2067
gi|63055057	beta-actin-like protein 2 [Homo sapiens]	5/5	1950
gi|3641621	gp180-carboxypeptidase D-like enzyme [Homo sapiens]	5/5	1937
gi|1841430	heavy neurofilament subunit [Homo sapiens]	5/5	1925
gi|4502249	arf-GAP with SH3 domain, ANK repeat and PH domain-containing protein 2 isoform a [Homo sapiens]	5/5	1881
gi|4529892	HSP70-2 [Homo sapiens]	5/5	1836
gi|316983160	NADH-ubiquinone oxidoreductase 75 kDa subunit, mitochondrial isoform 5 [Homo sapiens]	5/5	1814
gi|7656922	charged multivesicular body protein 2a [Homo sapiens]	5/5	1705
gi|4506775	membrane-bound transcription factor site-1 protease preproprotein [Homo sapiens]	5/5	1643
gi|15341178	lymphocyte alpha-kinase [Homo sapiens]	5/5	1630
gi|71664	calmodulin - salmon	5/5	1622
gi|6759917	A-kinase anchoring protein 18 gamma [Homo sapiens]	5/5	1508
gi|4520225	Rho kinase [Homo sapiens]	5/5	1456
gi|306891	90kDa heat shock protein [Homo sapiens]	5/5	1419
gi|4826659	F-actin-capping protein subunit beta isoform 1 [Homo sapiens]	5/5	1388
gi|1297274	beta-tubulin [Homo sapiens]	5/5	1295
gi|24308115	interferon regulatory factor 2-binding protein 1 [Homo sapiens]	5/5	1105
gi|194388088	unnamed protein product [Homo sapiens]	5/5	1111
gi|134133226	POTE ankyrin domain family member E [Homo sapiens]	5/5	1070
gi|62414289	vimentin [Homo sapiens]	5/5	988
gi|62897625	beta actin variant [Homo sapiens]	5/5	902
gi|190281	protein phosphatase I alpha subunit (PPPIA) (EC 3.1.3.16), partial [Homo sapiens]	5/5	814
gi|537906	autotaxin [Homo sapiens]	5/5	800
gi|4501881	actin, alpha skeletal muscle [Homo sapiens]	4/5	704

gi|193788501	unnamed protein product [Homo sapiens]	4/5	688
gi|435476	cytokeratin 9 [Homo sapiens]	4/5	640
gi|340021	alpha-tubulin [Homo sapiens]	4/5	623
gi|30908859	actin alpha 1 skeletal muscle protein [Homo sapiens]	4/5	591
gi|188586	myosin light chain 2 [Homo sapiens]	4/5	580
gi|4507729	tubulin beta-2A chain [Homo sapiens]	4/5	543
gi|35222	unnamed protein product [Homo sapiens]	4/5	500
gi|5729877	heat shock cognate 71 kDa protein isoform 1 [Homo sapiens]	4/5	477
gi|63055057	beta-actin-like protein 2 [Homo sapiens]	4/5	464
gi|145309046	POTE-2 alpha-actin [Homo sapiens]	4/5	395
gi|62089306	spectrin, alpha, non-erythrocytic 1 (alpha-fodrin) variant [Homo sapiens]	4/5	395
gi|10436240	unnamed protein product [Homo sapiens]	4/5	377
gi|31542947	60 kDa heat shock protein, mitochondrial [Homo sapiens]	4/5	316
gi|28317	unnamed protein product [Homo sapiens]	4/5	270
gi|189036	nonmuscle myosin heavy chain (NMHC) [Homo sapiens]	4/5	261
gi|61680528	Chain A, Trapped Intermediate Of Calmodulin [Homo sapiens]	4/5	213
gi|22415740	mutant desmin [Homo sapiens]	4/5	199
gi|307358	peripherin [Homo sapiens]	4/5	194
gi|32455244	serine/threonine-protein phosphatase 2A 65 kDa regulatory subunit A beta isoform isoform b [Homo sapiens]	4/5	193
gi|31092	unnamed protein product [Homo sapiens]	4/5	190
gi|1857526	beta-tubulin [Homo sapiens]	4/5	181
gi|5453740	myosin regulatory light chain 12A [Homo sapiens]	4/5	175
gi|538132	oxidoreductase [Homo sapiens]	4/5	175
gi|28334	unnamed protein product [Homo sapiens]	4/5	170
gi|1200072	keratin [Homo sapiens]	4/5	169
gi|338443	beta-spectrin [Homo sapiens]	4/5	168

gi|7959321	KIAA1527 protein [Homo sapiens]	4/5	168
gi|34365461	hypothetical protein [Homo sapiens]	4/5	165
gi|23441654	Kinesin-1 heavy chain [Homo sapiens]	3/5	160
gi|187957144	PHLDB3 protein [Homo sapiens]	3/5	160
gi|119623398	RAN binding protein 1, isoform CRA_f [Homo sapiens]	3/5	158
gi|192282133	phosphatidylinositol transfer protein, beta [Homo sapiens]	3/5	155
gi|2738149	kinesin-like motor protein KIF1C [Homo sapiens]	3/5	150
gi|288915525	BAG family molecular chaperone regulator 1 isoform BAG-1L [Homo sapiens]	3/5	150
gi|13540576	tudor domain-containing protein 3 isoform 2 [Homo sapiens]	3/5	145
gi|38540973	CCDC73 protein [Homo sapiens]	3/5	145
gi|4557373	biotinidase precursor [Homo sapiens]	3/5	142
gi|14043093	EMILIN1 protein [Homo sapiens]	3/5	140
gi|1518269	TGN46 [Homo sapiens]	3/5	136
gi|4757930	G2/mitotic-specific cyclin-B2 [Homo sapiens]	3/5	135
gi|29467442	cytosolic phospholipase A2 delta [Homo sapiens]	3/5	130
gi|386159	TLS-CHOP [Homo sapiens]	3/5	130
gi|4758220	XAP-5 protein [Homo sapiens]	3/5	130
gi|193785868	unnamed protein product [Homo sapiens]	3/5	130
gi|51491219	hypothetical protein [Homo sapiens]	3/5	127
gi|21624607	coactosin-like protein [Homo sapiens]	3/5	127
gi|28590	unnamed protein product [Homo sapiens]	3/5	126
gi|194385296	unnamed protein product [Homo sapiens]	3/5	126
gi|33469246	organic anion transporting polypeptide 8 [Homo sapiens]	3/5	126
gi|119602582	epiplakin 1 [Homo sapiens]	3/5	125
gi|145309046	POTE-2 alpha-actin [Homo sapiens]	3/5	125
gi|386758	GRP78 precursor [Homo sapiens]	3/5	125

gi|181573	cytokeratin 8 [Homo sapiens]	3/5	125
gi|63055057	beta-actin-like protein 2 [Homo sapiens]	3/5	125
gi|83318444	HSP90AA1 protein [Homo sapiens]	3/5	120
gi|181540	desmin [Homo sapiens]	3/5	120
gi|4758012	clathrin heavy chain 1 [Homo sapiens]	3/5	119
gi|6525069	tumor necrosis factor type 1 receptor ass	3/5	118
gi|31092	unnamed protein product [Homo sapiens]	3/5	110
gi|6690741	signal recognition particle 68 [Homo sapi	3/5	110
gi|340021	alpha-tubulin [Homo sapiens]	3/5	98
gi|338443	beta-spectrin [Homo sapiens]	3/5	98
gi|4757756	annexin A2 isoform 2 [Homo sapiens]	3/5	96
gi|17986258	myosin light polypeptide 6 isoform 1 [Homo sapiens]	3/5	96
gi|5453740	myosin regulatory light chain 12A [Homo sapiens]	3/5	96
gi|92091583	myosin-11 isoform SM2B [Homo sapiens]	3/5	96
gi|32111	unnamed protein product [Homo sapiens]	3/5	95
gi|1805280	alpha II spectrin [Homo sapiens]	3/5	95
gi|179106	nonerythroid alpha-spectrin [Homo sapiens]	3/5	94
gi|62421170	actin-like protein [Homo sapiens]	3/5	94
gi|340021	alpha-tubulin [Homo sapiens]	3/5	93
gi|24119203	tropomyosin alpha-3 chain isoform 2 [Homo sapiens]	3/5	93
gi|181573	cytokeratin 8 [Homo sapiens]	3/5	93
gi|158261511	unnamed protein product [Homo sapiens]	3/5	93
gi|2804273	alpha actinin 4 [Homo sapiens]	3/5	92
gi|31092	unnamed protein product [Homo sapiens]	3/5	92
gi|3901030	cytokeratin type II [Homo sapiens]	2/5	92
gi|32097	unnamed protein product [Homo sapiens]	2/5	90

gi|6573280	beta tropomyosin [Homo sapiens]	2/5	90
gi|119592246	myosin, heavy polypeptide 14, isoform CRA_c [Homo sapiens]	2/5	88
gi|194097350	alpha-actinin-1 isoform a [Homo sapiens]	2/5	88
gi|31074639	type I inner root sheath specific keratin 25 irs2 [Homo sapiens]	2/5	87
gi|34866	unnamed protein product [Homo sapiens]	2/5	87
gi|4507651	tropomyosin alpha-4 chain isoform 2 [Homo sapiens]	2/5	87
gi|6934244	tropomodulin 3 [Homo sapiens]	2/5	87
gi|179247	ADP/ATP carrier protein [Homo sapiens]	2/5	85
gi|9280816	myosin VI [Homo sapiens]	2/5	85
gi|9507215	tubulin alpha-8 chain isoform 1 [Homo sapiens]	2/5	85
gi|194381192	unnamed protein product [Homo sapiens]	2/5	85
gi|4557241	alpha-actinin-3 isoform 1 [Homo sapiens]	2/5	73
gi|119631384	hCG22236, isoform CRA_b [Homo sapiens]	2/5	73
gi|119631383	hCG22236, isoform CRA_a [Homo sapiens]	2/5	65
gi|119579661	hCG1748768, isoform CRA_b [Homo sapiens]	2/5	64
gi|14249342	alpha-internexin [Homo sapiens]	2/5	64
gi|3298502	xylulokinase [Homo sapiens]	2/5	64
gi|21740112	hypothetical protein [Homo sapiens]	2/5	62
gi|406854	cytokeratin 20 [Homo sapiens]	2/5	62
gi|467828	smooth muscle myosin alkali light chain [Homo sapiens]	2/5	55
gi|11065721	28kD interferon responsive protein [Homo sapiens]	2/5	54
gi|2627129	polyubiquitin [Homo sapiens]	2/5	50
gi|27529734	KIAA0727 protein [Homo sapiens]	2/5	50
gi|4506691	40S ribosomal protein S16 [Homo sapiens]	2/5	48
gi|190668	psoriasin [Homo sapiens]	2/5	48
gi|6005747	E3 ubiquitin-protein ligase RING2 [Homo sapiens]	2/5	48

gi|119625156	hCG2041078 [Homo sapiens]	2/5	45
gi|45768281	Histone cluster 1, H3i [Homo sapiens]	2/5	45
gi|21619965	CABP4 protein [Homo sapiens]	2/5	45
gi|56203471	histone cluster 2, H3, pseudogene 2 [Homo sapiens]	2/5	45
gi|33341788	PP7706 [Homo sapiens]	2/5	45
gi|10732604	unknown [Homo sapiens]	2/5	45
gi|94536840	transmembrane and coiled-coil domain-containing protein 6 [Homo sapiens]	2/5	45
gi|181965	elongation factor 1 alpha, partial [Homo sapiens]	2/5	44
gi|33333160	deubiquitinating enzyme DUB1 [Homo sapiens]	2/5	44
gi|28827782	zinc fingers and homeoboxes protein 3 [Homo sapiens]	2/5	44
gi|4507943	exportin-1 [Homo sapiens]	2/5	44
gi|119579994	hCG1812916, isoform CRA_c [Homo sapiens]	2/5	44
gi|7959223	KIAA1481 protein [Homo sapiens]	2/5	43
gi|10434147	unnamed protein product [Homo sapiens]	2/5	43
gi|40255260	PTB-containing, cubilin and LRP1-interacting protein isoform 1 [Homo sapiens]	2/5	42
gi|7959275	KIAA1507 protein [Homo sapiens]	2/5	42
gi|84683052	immunoglobulin heavy chain variable region, partial [Homo sapiens]	2/5	40
gi|12053261	hypothetical protein [Homo sapiens]	2/5	40
gi|14042481	unnamed protein product [Homo sapiens]	2/5	39
gi|8489859	26S proteasome-associated UCH interacting protein 1 [Homo sapiens]	2/5	39
gi|34531104	unnamed protein product [Homo sapiens]	2/5	39
gi|4826914	JMJD7-PLA2G4B protein isoform a [Homo sapiens]	2/5	39
gi|119592756	transmembrane protein 86B, isoform CRA_a [Homo sapiens]	2/5	38
gi|3169653	vesicle transport related protein [Homo sapiens]	2/5	37
gi|641958	non-muscle myosin B [Homo sapiens]	2/5	37
gi|30841835	EGF domain-containing protein [Homo sapiens]	2/5	36

gi|158254450	unnamed protein product [Homo sapiens]	2/5	35
gi|4507181	leukosialin precursor [Homo sapiens]	2/5	35
gi|10437774	unnamed protein product [Homo sapiens]	2/5	35
gi|29150700	myeloid/lymphoid or mixed-lineage leukemia 5 [Homo sapiens]	2/5	34
gi|194385468	unnamed protein product [Homo sapiens]	2/5	34
gi|14388939	chorea-acanthocytosis [Homo sapiens]	2/5	33
gi|119592756	transmembrane protein 86B, isoform CRA_a [Homo sapiens]	2/5	33
gi|184039	sodium channel alpha subunit [Homo sapiens]	2/5	33
gi|148733176	ubiquitously transcribed tetratricopeptide repeat protein Y-linked transcript variant 66 [Homo sapiens]	2/5	32
gi|10436970	unnamed protein product [Homo sapiens]	2/5	32
gi|13375848	structural maintenance of chromosomes protein 6 [Homo sapiens]	2/5	32
gi|119631344	FLJ44048 protein, isoform CRA_a [Homo sapiens]	2/5	32
gi|28592	serum albumin [Homo sapiens]	2/5	32
gi|10434106	unnamed protein product [Homo sapiens]	2/5	32
gi|119628465	hCG1646195 [Homo sapiens]	2/5	32
gi|119608065	GTPase activating Rap/RanGAP domain-like 3, isoform CRA_f [Homo sapiens]	2/5	32
gi|16549885	unnamed protein product [Homo sapiens]	2/5	32
gi|21758373	unnamed protein product [Homo sapiens]	2/5	32
gi|1480787	transaldolase	2/5	32

Supplementary Table 2

ID	Gene name	Symbol	FL (1244)	Nt (Δ1-622)	Nt1 (Δ1-271)	Nt2 (Δ249-569)	Mid (Δ431-681)	Ct (Δ960-1244)	Total
12667788	myosin-9 [Homo sapiens]	MYH9	3	3	3	2	0	2	13
3641621	gp180-carboxypeptidase D-like enzyme [Homo sapiens]	CPD	3	2	2	1	2	2	12
1841430	heavy neurofilament subunit [Homo sapiens]	NEFH	2	1	2	0	2	3	10
119608065	GTPase activating Rap/RanGAP domain-like 3, isoform CRA_f [Homo sapiens]	GARNL3	2	1	2	1	2	2	10
28195394	histone H2A type 2-B [Homo sapiens]	HIST2H2AB	2	1	1	1	2	2	9
19072649	TPMsk3 [Homo sapiens]	TPM3	1	1	1	2	1	2	8
4826659	F-actin-capping protein subunit beta isoform 1 [Homo sapiens]	CAPZB	3	3	1	0	0	0	7
537906	autotaxin [Homo sapiens]	ENPP2	2	0	1	1	2	1	7
2981235	mitotic checkpoint kinase Mad3L [Homo sapiens]	BUB1B	1	1	2	1	2	0	7
4502249	arf-GAP with SH3 domain, ANK repeat and PH domain-containing protein 2 isoform a [Homo sapiens]	ASAP1	2	2	1	1	0	1	7
6626179	bromodomain protein CELTIX1 [Homo sapiens]	BRD7	1	0	2	0	2	2	7
28376621	SEC14-like protein 4 isoform a [Homo sapiens]	SEC14L4	0	1	2	1	2	1	7
83674986	rcTPM3 [Homo sapiens]	TPM3	1	1	1	0	2	2	7
316983160	NADH-ubiquinone oxidoreductase 75 kDa subunit, mitochondrial isoform 5 [Homo sapiens]	NDUFS1	2	1	0	1	0	3	7
4929625	CGI-77 protein [Homo sapiens]	OTUD6B	1	2	2	0	1	0	6
5453740	myosin regulatory light chain 12A [Homo sapiens]	MYL12A	2	0	1	1	1	1	6
7656922	charged multivesicular body protein 2a [Homo sapiens]	CHMP2A	2	0	1	1	1	1	6
71664	calmodulin - salmon	CAM1	2	2	2	0	0	0	6
134133226	POTE ankyrin domain family member E [Homo sapiens]	POTEE	1	1	2	0	1	1	6
178027	alpha-actin [Homo sapiens]	ACTA1	1	1	0	1	1	1	5
4504251	histone H2A type 2-A [Homo sapiens]	HIST2H2AA3	1	0	1	1	1	1	5
4506775	membrane-bound transcription factor site-1 protease preproprotein [Homo sapiens]	MBTPS1	1	1	1	1	0	1	5

4757910	cocaine- and amphetamine-regulated transcript protein preproprotein [Homo sapiens]	CARTPT	1	1	0	1	1	1	5
42300771	Ras-related protein Rab-11A	RAB11A	2	1	0	0	1	1	5
6934244	tropomodulin 3 [Homo sapiens]	TMOD3	2	0	1	1	1	0	5
8923398	cell growth-regulating nucleolar protein [Homo sapiens]	LYAR	1	1	1	1	0	1	5
15341178	lymphocyte alpha-kinase [Homo sapiens]	ALPK1	3	2	0	0	0	0	5
31979	histone H2A.2 [Homo sapiens]	HTA2	2	0	0	1	1	0	4
1212917	axonal transporter of synaptic vesicles [Homo sapiens]	KIF1A	0	1	1	0	1	1	4
2605501	OCTN1 [Homo sapiens]	SLC22A4	1	0	1	1	1	0	4
6331301	KIAA1276 protein [Homo sapiens]	FAM184B	0	1	1	0	1	1	4
8923110	nuclear distribution protein nudE homolog 1 [Homo sapiens]	NDE1	1	1	0	1	0	1	4
9507215	tubulin alpha-8 chain isoform 1 [Homo sapiens]	TUBA8	1	0	1	1	1	0	4
14249342	alpha-internexin [Homo sapiens]	INA	1	0	1	0	1	1	4
21450887	UBE3B variant 1 [Homo sapiens]	UBE3B	0	1	1	1	1	0	4
28703800	SLC25A28 protein, partial [Homo sapiens]	SLC25A28	1	1	1	0	1	1	4
158937236	puromycin-sensitive aminopeptidase [Homo sapiens]	NPEPPS	2	1	0	1	0	0	4
29421178	KIAA0591 protein [Homo sapiens]	KIF1B	0	0	2	0	2	0	4
181540	desmin [Homo sapiens]	DES	1	0	0	0	1	1	3
188586	myosin light chain 2 [Homo sapiens]	MYL2	0	0	1	1	1	0	3
447224	vasopressin receptor:ISOTYPE=V2	AVPR2	1	0	0	0	1	1	3
1654344	guanine nucleotide exchange factor p115-RhoGEF [Homo sapiens]	ARHGEF1	1	0	0	1	0	1	3
23441654	Kinesin-1 heavy chain [Homo sapiens]	KIF5B	0	0	0	0	1	2	3
2104553	Myosin heavy chain (MHY11) (5'partial) [Homo sapiens]	MYH11	0	0	1	1	1	0	3
2465540	phosphodiesterase I/nucleotide pyrophosphatase beta [Homo sapiens]	ENPP3	0	1	1	0	1	0	3
4504255	histone H2A.Z [Homo sapiens]	H2AFZ	2	0	0	0	0	1	3
4586460	tudor repeat associator with PCTAIRE 2 [Homo sapiens]	TDRD7	1	0	0	1	1	0	3
4885377	histone H1.3 [Homo sapiens]	HIST1H1D	0	0	0	0	1	2	3

6759917	A-kinase anchoring protein 18 gamma [Homo sapiens]	AKAP7	1	0	0	0	1	1	3
14043093	EMILIN1 protein [Homo sapiens]	EMILIN1	1	0	1	0	0	1	3
86371123	autogenous vein graft remodeling associated protein 2 [Homo sapiens]	AVGR2	0	0	1	0	1	1	3
153791352	POTE ankyrin domain family member F [Homo sapiens]	POTEF	1	0	0	1	0	1	3
4507729	tubulin beta-2A chain [Homo sapiens]	TUBB2A	1	0	0	2	0	0	3
30506	desmoglein type 1 [Homo sapiens]	DSG1	0	0	1	0	0	1	2
179106	nonerythroid alpha-spectrin [Homo sapiens]	SPTAN1	1	0	1	0	0	0	2
190281	protein phosphatase I alpha subunit (PPPIA) (EC 3.1.3.16), partial [Homo sapiens]	PPP1CA	1	0	0	0	0	1	2
226726	transcription factor ATF1	AFT1	0	2	0	0	0	0	2
392890	drebrin E2 [Homo sapiens]	DBN1	0	0	0	1	0	1	2
1805280	alpha II spectrin [Homo sapiens]	SPTAN1	1	0	1	0	0	0	2
1871168	sodium channel 2 [Homo sapiens]	SCN2A	0	0	1	0	1	0	2
1911650	alpha,alpha-trehalase {EC 3.2.1.28} [human, kidney, Peptide Partial, 308 aa]	TREH	1	0	1	0	0	0	2
2843123	alpha tubulin [Homo sapiens]	TUBA1A	0	0	1	1	0	0	2
3298502	xylulokinase [Homo sapiens]	XYLB	0	0	0	1	0	1	2
4240313	KIAA0912 protein [Homo sapiens]	CEP152	0	0	1	0	1	0	2
4503979	glial fibrillary acidic protein isoform 1 [Homo sapiens]	GFAP	0	0	1	0	0	1	2
4520225	Rho kinase [Homo sapiens]	ROCK1	0	1	1	0	0	0	2
5453597	F-actin-capping protein subunit alpha-1 [Homo sapiens]	CAPZA1	0	0	1	0	1	0	2
6470129	cancer-amplified transcriptional coactivator ASC-2 [Homo sapiens]	NCOA6	0	0	0	1	1	0	2
6648146	jdp2 [Homo sapiens]	JDP2	0	0	0	0	1	1	2
6841138	HSPC099, partial [Homo sapiens]	RPRD2	0	0	0	0	1	1	2
15079530	GPNMB protein [Homo sapiens]	GPNMB	1	0	0	1	0	0	2
15823653	ALS2CR12 [Homo sapiens]	ALS2CR12	0	1	0	1	0	0	2
16877475	PPIL4 protein [Homo sapiens]	PPIL4	1	0	0	0	1	0	2
29171306	solute carrier family 13 member 5 isoform a [Homo sapiens]	SLC13A5	0	2	0	0	0	0	2

32455244	serine/threonine-protein phosphatase 2A 65 kDa regulatory subunit A beta isoform isoform b [Homo sapiens]	PPP2R1B	1	0	0	0	0	1	2
40789062	KIAA0055 [Homo sapiens]	USP8	0	1	0	0	1	0	2
47939651	KRT17 protein [Homo sapiens]	KRT17	0	0	0	1	1	0	2
48146275	H2AFX [Homo sapiens]	H2AFX	0	0	0	1	0	1	2
55960095	dystonin [Homo sapiens]	DST	1	0	1	0	0	0	2
62088056	tensin variant [Homo sapiens]	TNS1	0	0	1	0	0	1	2
68533107	MYH10 variant protein [Homo sapiens]	MYO10	1	0	0	0	0	1	2
119631362	NCK-associated protein 1, isoform CRA_f [Homo sapiens]	NCKAP1	1	1	0	0	0	0	2
119631808	bromodomain adjacent to zinc finger domain, 2B, isoform CRA_a [Homo sapiens]	BAZ2A	1	0	0	1	0	0	2
148733176	ubiquitously transcribed tetratricopeptide repeat protein Y-linked transcript variant 66 [Homo sapiens]	UTY	0	0	0	1	0	1	2
149773468	RAD51-associated protein 2 [Homo sapiens]	RAD51AP2	1	0	0	1	0	0	2
221219020	WD repeat-containing protein 87 [Homo sapiens]	WDR87	0	0	0	0	1	1	2
34812	utrophin (dystrophin related protein) [Homo sapiens]	UTRN	0	1	0	0	0	0	1
35046	NF-M [Homo sapiens]	NEFM	1	0	0	0	0	0	1
179734	plasma membrane calcium ATPase isoform 2 [Homo sapiens]	ATP2B2	0	0	0	0	1	0	1
190668	psoriasin [Homo sapiens]	S100A7	0	0	0	0	0	1	1
242988	lipoprotein lipase [Homo sapiens]	LPL	0	0	0	0	1	0	1
248842	aggrecan core protein [human, synovial fluid, Peptide Partial, 11 aa]	ACAN	0	1	0	0	0	0	1
306702	dehydroepiandrosterone sulfotransferase [Homo sapiens]	SULT2A1	0	1	0	0	0	0	1
386991	oxytocin-neurophysin I [Homo sapiens]	OXT	0	0	0	0	0	1	1
473431	microtubule-associated protein 1B [Homo sapiens]	MAP1B	1	0	0	0	0	0	1
529662	ataxin-1 [Homo sapiens]	ATXN1	0	0	0	1	0	0	1
780806	p21-activated protein kinase [Homo sapiens]	PAK1	0	0	0	0	1	0	1
790538	GluR4 [Homo sapiens]	GRIA4	0	0	0	0	0	1	1
791004	ARSE [Homo sapiens]	ARSE	1	0	0	0	0	0	1
994844	neurofilament-66 [Homo sapiens]	INA	0	0	0	0	1	0	1

1147813	desmoplakin I [Homo sapiens]	DSP	0	0	0	0	0	1	1
1161384	BRCA2 [Homo sapiens]	BRCA2	1	0	0	0	0	0	1
1246749	biliverdin IX alpha reductase [Homo sapiens]	BLVRA	1	0	0	0	0	0	1
1279504	NF-L [Homo sapiens]	NEFL	1	0	0	0	0	0	1
1381142	Hs-CUL-1 [Homo sapiens]	CUL1	1	0	0	0	0	0	1
1480787	transaldolase [Homo sapiens]	TALDO1	1	0	0	0	0	0	1
1888354	placental leucine aminopeptidase [Homo sapiens]	LNPEP	0	0	0	1	0	0	1
1934601	I kappa B epsilon [Homo sapiens]	NFKBIE	0	1	0	0	0	0	1
2224539	KIAA0299 [Homo sapiens]	DOCK3	0	0	1	0	0	0	1
2224677	KIAA0368 [Homo sapiens]	KIAA0368	0	0	0	0	0	1	1
2230871	Miz-1 protein [Homo sapiens]	ZBTB17	0	0	0	0	1	0	1
2467369	SCP-1 [Homo sapiens]	SYCP1	0	0	1	0	0	0	1
2738149	kinesin-like motor protein KIF1C [Homo sapiens]	KIF1C	0	0	0	0	0	1	1
3002951	breakpoint cluster region protein 1 [Homo sapiens]	BCR	0	0	0	0	0	1	1
3043596	KIAA0536 protein [Homo sapiens]	PRPF4B	1	0	0	0	0	0	1
3327094	KIAA0640 protein [Homo sapiens]	SWAP70	0	0	0	1	0	0	1
3327212	KIAA0699 protein [Homo sapiens]	BICD2	0	0	0	0	1	0	1
3360457	cul-3 [Homo sapiens]	CUL3	1	0	0	0	0	0	1
3789765	transmembrane receptor UNC5C [Homo sapiens]	UNC5C	0	1	0	0	0	0	1
3925685	PIBF1 protein [Homo sapiens]	PIBF1	0	0	0	0	1	0	1
4504007	glycerol kinase isoform b [Homo sapiens]	GK	0	0	0	1	0	0	1
4504335	kalirin isoform 2 [Homo sapiens]	KALRN	1	0	0	0	0	0	1
4505317	protein phosphatase 1 regulatory subunit 12A isoform a [Homo sapiens]	PPP1R12A	0	0	1	0	0	0	1
4505997	protein phosphatase 1D [Homo sapiens]	PPM1D	0	0	0	1	0	0	1
4506619	60S ribosomal protein L24 [Homo sapiens]	RPL24	0	0	0	1	0	0	1
4506691	40S ribosomal protein S16 [Homo sapiens]	RPS16	0	0	0	0	0	1	1

4506773	protein S100-A9 [Homo sapiens]	S100A9	1	0	0	0	0	0	1
4506813	sodium channel protein type 9 subunit alpha [Homo sapiens]	SCN9A	0	0	0	0	0	1	1
4507357	transgelin-2 [Homo sapiens]	TAGLN2	0	0	0	1	0	0	1
4507369	tyrosine aminotransferase [Homo sapiens]	TAT	0	0	0	1	0	0	1
4557303	fatty aldehyde dehydrogenase isoform 2 [Homo sapiens]	ALDH3A2	0	0	0	1	0	0	1
4557565	DNA excision repair protein ERCC-6 [Homo sapiens]	ERCC6	0	0	1	0	0	0	1
4757930	G2/mitotic-specific cyclin-B2 [Homo sapiens]	CCNB2	0	1	0	0	0	0	1
4826760	heterogeneous nuclear ribonucleoprotein F [Homo sapiens]	HNRNPH1	0	0	0	1	0	0	1
4835900	Mad2B protein [Homo sapiens]	MAD2L2	1	0	0	0	0	0	1
4885489	proto-oncogene serine/threonine-protein kinase mos [Homo sapiens]	MOS	0	0	0	0	0	1	1
4894717	3',5'-cyclic nucleotide phosphodiesterase 10A2 [Homo sapiens]	PDE10A	1	0	0	0	0	0	1
5032123	protein SSX4 isoform a [Homo sapiens]	SSX4	1	0	0	0	0	0	1
5360204	A-kinase anchor protein [Homo sapiens]	AKAP8	0	0	0	0	1	0	1
5689389	KIAA1026 protein [Homo sapiens]	KIAA1026	1	0	0	0	0	0	1
5689413	KIAA1038 protein [Homo sapiens]	HBS1L	0	0	0	0	1	0	1
5732237	malonyl coenzyme A decarboxylase [Homo sapiens]	MLYCD	0	0	0	1	0	0	1
6005747	E3 ubiquitin-protein ligase RING2 [Homo sapiens]	RNF2	0	0	0	0	0	1	1
6330700	KIAA1229 protein [Homo sapiens]	ODF2L	0	1	0	0	0	0	1
6539606	metastasis suppressor protein [Homo sapiens]	MTSS1	0	1	0	0	0	0	1
7105926	calcium channel alpha2-delta3 subunit [Homo sapiens]	CACNA2D3	0	0	0	0	1	0	1
7662254	hephaestin isoform b [Homo sapiens]	HEPH	1	0	0	0	0	0	1
7739737	G-protein-coupled receptor 48 [Homo sapiens]	GPR88	0	0	0	1	0	0	1
7770257	PRO2964 [Homo sapiens]	PRO2964	0	1	0	0	0	0	1
9844110	cAMP-specific phosphodiesterase 4D [Homo sapiens]	PDE4D	0	1	0	0	0	0	1
12698003	KIAA1729 protein [Homo sapiens]	ZNF518B	0	0	0	1	0	0	1
13177775	Minichromosome maintenance complex component 5 [Homo sapiens]	MCMBP	0	0	1	0	0	0	1

13277572	KIAA1632 protein, partial [Homo sapiens]	KIAA1632	0	0	1	0	0	0	1
13365515	scavenger receptor with C-type lectin type I [Homo sapiens]	COLEC12	0	1	0	0	0	0	1
13569960	interferon-stimulated 20 kDa exonuclease-like 2 [Homo sapiens]	ISG20L2	1	0	0	0	0	0	1
14043107	FSCN1 protein [Homo sapiens]	FSCN1	0	0	1	0	0	0	1
14150139	partner of Y14 and mago isoform 1 [Homo sapiens]	WIBG	0	0	0	0	0	1	1
14250636	Nuclear factor of kappa light polypeptide gene enhancer in B-cells inhibitor-like 2 [Homo sapiens]	TONSL	1	0	0	0	0	0	1
15079708	GPRIN2 protein [Homo sapiens]	GPRIN2	1	0	0	0	0	0	1
15559516	Acyl-CoA synthetase family member 2 [Homo sapiens]	ACSF2	0	0	0	1	0	0	1
17066105	Titin [Homo sapiens]	TTN	0	0	0	0	0	1	1
18088559	PYHIN1 protein [Homo sapiens]	PYHIN1	0	0	0	1	0	0	1
18916721	KIAA1940 protein [Homo sapiens]	FBXO41	0	0	1	0	0	0	1
19702127	P-Rex1 protein [Homo sapiens]	PREX1	0	0	1	0	0	0	1
20304127	patatin-like phospholipase domain-containing protein 5 isoform 1 [Homo sapiens]	PNPLA1	0	0	1	0	0	0	1
20521766	KIAA1102 protein [Homo sapiens]	LIMCH1	0	0	0	0	1	0	1
20521884	KIAA1327 protein [Homo sapiens]	BOD1L1	0	0	0	0	0	1	1
21450705	leucine-rich repeat-containing protein 45 [Homo sapiens]	LRRC45	0	0	0	1	0	0	1
21542532	LGR4 protein [Homo sapiens]	LGR4	0	0	1	0	0	0	1
21619965	CABP4 protein [Homo sapiens]	CABP4	0	0	0	0	0	1	1
21666316	solute carrier family 12 member 8 [Homo sapiens]	SLC12A8	0	0	1	0	0	0	1
22651773	vanilloid receptor like 3 protein splice variant b [Homo sapiens]	TRPV4	0	0	1	0	0	0	1
23397570	PI-PLC X domain-containing protein 2 isoform b [Homo sapiens]	PLCXD2	1	0	0	0	0	0	1
23958561	REST corepressor 2 [Homo sapiens]	RCOR2	0	0	0	0	1	0	1
24308115	interferon regulatory factor 2-binding protein 1 [Homo sapiens]	IRF2BP1	1	0	0	0	0	0	1
26986023	AP20 region protein [Homo sapiens]	APRG1	0	1	0	0	0	0	1
27451602	tau-tubulin kinase [Homo sapiens]	TTBK1	0	0	0	0	0	1	1
28827782	zinc fingers and homeoboxes protein 3 [Homo sapiens]	ZHX1	0	0	0	0	0	1	1

29150700	myeloid/lymphoid or mixed-lineage leukemia 5 [Homo sapiens]	MLL5	0	0	0	0	0	1	1
31542781	nuclear envelope phosphatase-regulatory subunit 1 [Homo sapiens]	CNEP1R1	0	0	1	0	0	0	1
32450527	CCDC25 protein, partial [Homo sapiens]	CCDC25	0	0	0	0	1	0	1
33333160	deubiquitinating enzyme DUB1 [Homo sapiens]	DUB1	0	0	0	0	0	1	1
33519481	neural cell adhesion molecule 2 precursor [Homo sapiens]	CDON	1	0	0	0	0	0	1
34147416	breast cancer metastasis-suppressor 1-like protein [Homo sapiens]	BRMS1L	0	0	0	1	0	0	1
34192567	D-2-hydroxyglutarate dehydrogenase [Homo sapiens]	D2HGDH	1	0	0	0	0	0	1
34223761	diaphanous 1 [Homo sapiens]	DIAPH1	1	0	0	0	0	0	1
37537696	M-phase phosphoprotein 9 [Homo sapiens]	MPHOSPH9	0	0	0	1	0	0	1
39652628	constitutive coactivator of PPAR-gamma-like protein 1 [Homo sapiens]	FAM120A	0	1	0	0	0	0	1
40255260	PTB-containing, cubilin and LRP1-interacting protein isoform 1 [Homo sapiens]	PID1	0	0	0	0	1	0	1
40788215	KIAA0331 [Homo sapiens]	SEMA3E	0	0	1	0	0	0	1
40788297	KIAA0590 protein [Homo sapiens]	IFT140	0	1	0	0	0	0	1
41350212	bromodomain-containing protein 7 isoform 2 [Homo sapiens]	BRD7	0	0	0	1	0	0	1
45504346	olfactory receptor 2K2 [Homo sapiens]	OR2K2	0	0	0	0	1	0	1
45592963	ras-like protein family member 11A [Homo sapiens]	RASL11A	0	0	0	0	1	0	1
46240864	sperm-associated antigen 17 [Homo sapiens]	SPAG7	0	0	0	0	1	0	1
50295493	MCTP1L [Homo sapiens]	MCTP1	0	1	0	0	0	0	1
50949258	hHa8 protein [Homo sapiens]	HSP70h	1	0	0	0	0	0	1
55663144	Ral GEF with PH domain and SH3 binding motif 2 [Homo sapiens]	RALGPS2	1	0	0	0	0	0	1
56203471	histone cluster 2, H3, pseudogene 2 [Homo sapiens]	HIST2H3PS2	0	0	0	0	0	1	1
57157125	G-protein coupled receptor [Homo sapiens]	GPR4	0	0	0	1	0	0	1
62122917	filaggrin-2 [Homo sapiens]	FLG2	0	0	0	0	0	1	1
62898131	pantothenate kinase 3 variant [Homo sapiens]	PANK3	0	0	0	0	0	1	1
71061468	centromere-associated protein E [Homo sapiens]	CENPE	0	0	0	0	0	1	1
82697315	protein RUFY3 isoform 1 [Homo sapiens]	RUFY3	0	0	1	0	0	0	1

87299628	biorientation of chromosomes in cell division protein 1-like [Homo sapiens]	BOD1L1	0	0	0	0	1	0	1
94536840	transmembrane and coiled-coil domain-containing protein 6 [Homo sapiens]	TMCO6	0	0	0	0	0	1	1
114520607	E3 ubiquitin-protein ligase NEDD4 isoform 2 [Homo sapiens]	NEDD4	0	0	0	1	0	0	1
117558503	KRT83 protein [Homo sapiens]	KRT83	0	0	0	0	0	1	1
119568103	F-box protein 5, isoform CRA_a [Homo sapiens]	FBXO5	0	0	1	0	0	0	1
119571419	kinase suppressor of ras 1, isoform CRA_a [Homo sapiens]	KSR1	0	0	0	0	0	1	1
119589483	tumor necrosis factor (ligand) superfamily, member 9, isoform CRA_b [Homo sapiens]	TNFSF9	0	0	0	1	0	0	1
119605398	drebrin 1, isoform CRA_d [Homo sapiens]	DBN1	0	0	1	0	0	0	1
154813199	poly [ADP-ribose] polymerase 14 [Homo sapiens]	PARG	0	0	0	0	0	1	1
187957144	PHLDB3 protein [Homo sapiens]	PHLDB3	0	0	0	0	0	1	1
189031305	autotaxin isoform gamma [Homo sapiens]	ENPP2	0	1	0	0	0	0	1
338221674	disks large-associated protein 1 isoform 4 [Homo sapiens]	DLGAP5	0	0	0	0	1	0	1
371779716	truncated CADM1 protein [Homo sapiens]	CADM1	0	0	1	0	0	0	1
378925642	E3 ubiquitin-protein ligase RNF34 isoform 3 [Homo sapiens]	RNF34	0	1	0	0	0	0	1

Supplementary Table 3. Clinical characteristics of study population

Male subjects	Disease	Age (years)	Uric acid (mg/dL)	Creatinine (mg/dL)	GFR (%)	Benzbromarone treatment	Colchicine treatment
1	gout	31	5.3	1.03	84	+	+
2	gout	47	6.1	1.29	59	+	+
3	gout	52	5.4	1.04	75	+	+
4	gout	57	5.2	0.95	81	+	+
5	gout	61	4.0	1.14	65	+	+
6	gout	62	8.4	1.88	36	+	+
7	gout	69	7.5	1.15	63	+	+
8	gout	77	4.8	1.09	65	+	+
9	gout	24	10.7	1.18	75	−	−
10	gout	27	10.7	1.00	89	−	−
11	gout	33	7.6	0.86	102	−	−
12	gout	33	4.0	1.33	62	−	−
13	gout	35	7.7	0.86	101	−	−
14	gout	41	6.4	0.86	98	−	−
15	gout	42	10.2	1.13	71	−	−
16	gout	51	10.4	0.93	85	−	−
17	gout	59	15.8	2.18	31	−	−
18	gout	60	6.7	1.02	74	−	−
19	gout	62	9.1	1.37	52	−	−
20	gout	81	5.2	1.19	59	−	−
1	control	39	5.1				
2	control	40	6.7				
3	control	45	7				
4	control	46	2.8				
5	control	47	7.2				
6	control	51	6.0				
7	control	59	5.2				
8	control	66	6.0				
9	control	71	5.5				
10	control	75	6.4				

*Qd (every day); —, without treatment before enrolled in the study

Supplementary Figure 1

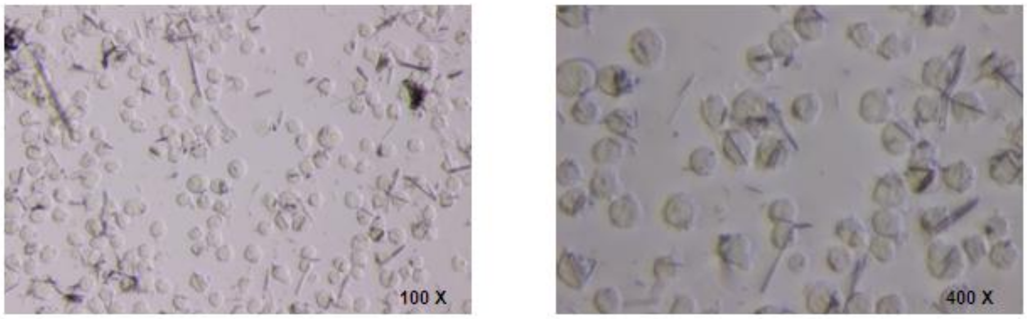

Supplementary Figure 2

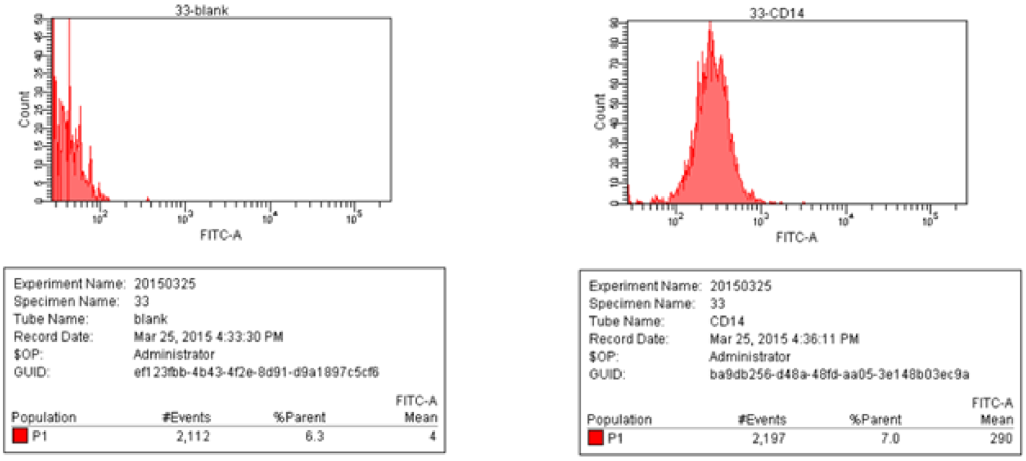

Supplementary Figure 3

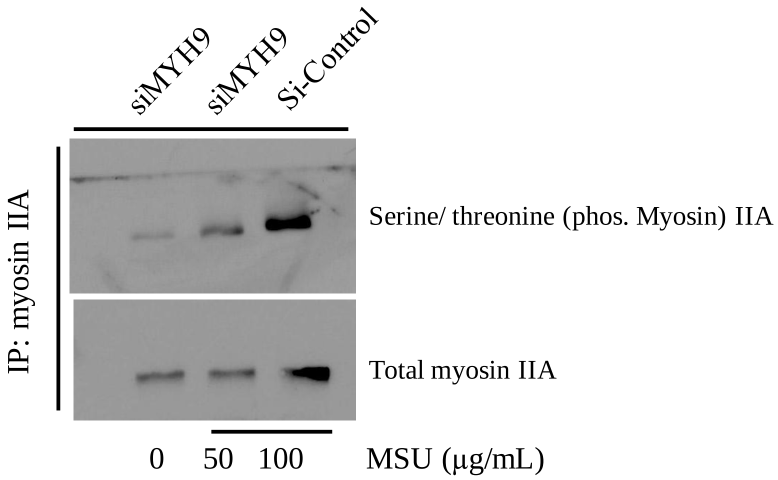

Supplementary Table 1

Over 173 candidate proteins for interaction passed at least two out of five independent experiments. Proteins are listed from the highest to lowest score (the ions score is −10*Log (P), where P is the probability that the observed match is a random event, individual ions scores of >32 indicate identity or extensive homology $p < 0.05$). Trypsin-digested eluates were subjected to LC/MS/MS peptide identification.

Supplementary Table 2

Total 218 proteins candidates were observed in at least one of three independent experiments; the cumulative amount of interacting protein dataset was detected by precipitating each serial deletion from protein using LC/MS/MS peptide identification. The list was arranged based on cumulative frequency from the highest to lowest.

Supplementary Table 3

Clinical characteristics of the study population

Supplementary Figure 1

THP-1 cells were treated with MSU crystals, and observed under inverted microscopy

Supplementary Figure 2

Purity of isolated monocytes was >80% validated to express CD14 by flow cytometry (FACS)

Supplementary Figure 3

Myosin IIA phosphorylation increased with MSU treatment (0 μg/mL versus 50 μg/mL) when MYH9 was knocked down (lane 1 and 2). The efficiency of si-MYH9 was not complete, refer to Fig 5A. A control without knockdown and high MSU (100 μg/mL) in lane 3 showed the highest level of myosin IIA phosphorylation.
